# The Unrealized Potential of Addiction Science in Curbing the HIV Epidemic

**DOI:** 10.2174/157016211798038605

**Published:** 2011-09

**Authors:** Nora D Volkow, Ruben D Baler, Jacques L Normand

**Affiliations:** National Institute on Drug Abuse, National Institutes of Health, Bethesda, Maryland, USA

**Keywords:** HAART, highly active antiretroviral therapy, HIV prevention, substance use disorders, substance use disorders treatment.

## Abstract

The stubbornly high incidence of new HIV infections belies the overwhelming evidence showing that sustained highly active antiretroviral therapy (HAART) has the power to dramatically reduce the spread of HIV infection and forever change the face of this devastating epidemic. One of the main contributors to this public health paradox is the ongoing HIV epidemic among substance users who contribute significantly to HIV infection rates through injection drug use and high-risk sexual behaviours. Current evidence clearly shows that, in order to fill this gap, we need to integrate substance abuse treatment with HIV treatment programmes and provide substance abusers with universal access to HIV treatment through a focussed effort to seek, test, treat, and retain hard-to-reach high risk individuals. These aims will require structural changes in the health care system to overcome many of the obstacles that have inhibited the merging of substance abuse treatment with HIV programmes for far too long.

As this special issue demonstrates, there is now ample evidence that sustained highly active antiretroviral therapy (HAART) leads to significant reductions in the spread of Human Immunodeficiency Virus (HIV) infection. Vigorous deployment of HAART has practically eliminated vertical HIV transmission in the developed world and even curtailed horizontal transmission among serodiscordant heterosexual couples. And expansion of HAART coverage, particularly among high risk populations, further reduces HIV transmission [1].

However, the incidence of new infections - estimated at about 55,400 per year in the past decade - has failed to reflect HAART’s clinical efficacy and promising outlook [2]. The main drivers for new HIV transmissions are undetected seropositive individuals: 1 in 5 of those infected in the United States (US) is unaware of his/her HIV positive status. A second contributor is the ongoing HIV epidemic among substance users, not just intravenous, but also non-intravenous drug users. Non-injecting drug users (IDUs) contribute significantly to HIV infection rates, not only because they share social and sexual networks with those of IDUs, but also because of their strong likelihood of engaging in high-risk sexual behaviours [3].

Addiction research has uncovered critical information about the impact of substance abuse behaviours on the risk of HIV transmission by shedding light on the acute and long-term deleterious effects of drugs of abuse on cognitive control, particularly as they interfere with normal frontocortical activity. It has been known for over two decades that the rewarding effects of drugs abused by humans are associated with increases in dopamine (DA) in the nucleus accumbens (NAc), which is a central hub in the brain’s reward network [4]. Not surprisingly, sex also increases DA in the NAc. Since DA increases in the NAc result in arousal and activation, the drug-induced DA increases could enhance the motivation to engage in sexual behaviours [5]. Indeed, preclinical and clinical studies have shown that drug-induced intoxication can enhance sexual behaviours. For example, in female rats, proceptivity (a measure of motivation for sexual behaviour) is enhanced by methamphetamine (MA) [6], and in humans, intravenous methylphenidate (a stimulant drug) has been shown to increase sexual arousal both in controls and in cocaine abusers [7]. Brain imaging studies have also shown that during intoxication there is a concomitant inhibition of areas involved with cognitive control. For example, with alcohol intoxication there is not only an increased activation of limbic brain regions implicated in sexual arousal (NAc and amygdala) but also an inhibition of brain regions involved with cognitive control (prefrontal cortex and cingulate gyrus) [8]. These findings are consistent with the reduced control and impulsivity reported during alcohol intoxication [9] and may foreshadow the increased loss of control that characterises addiction. In fact, it has been proposed that, during the establishment of an addiction, a more entrenched functional imbalance emerges between an over-reactive amygdala and a weakened prefrontal cortex that has a direct, negative impact on decision making and impulse control [10]. Brain imaging studies support the notion that emotional self-regulation (such as seen during sexual arousal) is implemented by a neural circuitry comprising various prefrontal and subcortical limbic regions [11]. Furthermore, dopaminergic striato-cortical pathways play a critical role in orchestrating the balanced communication required to mount proper decision making and keep impulsivity in check. In this regard the significantly lower level of striatal DA D2 receptors, which is consistently observed in addicted subjects, has been linked with higher scores in impulsivity [12] and with impaired frontal activity [13].

Knowledge of the connections between substance abuse and HIV transmission, whether through direct (contaminated needles) or indirect (increased impulsivity leading to high risk behaviours) means should be a powerful incentive for incorporating HIV prevention and treatment in substance abusing populations [14]. Unfortunately, broad application of this rationale is hampered by several obstacles. First and foremost is the reluctance to initiate HAART in substance abusers. This reluctance has been sustained by the belief that substance abusers are unable to adhere to HAART, compromising treatment effectiveness and promoting the emergence of drug-resistant HIV strains [15]. However, recent evidence demonstrates that after adjusting for adherence, drug-injecting substance users and nonusers have comparable 5-year survival when receiving HAART [16]. Also, out-of-treatment IDUs inject drugs, share needles, visit shooting galleries, and practise unsafe sex at significantly higher rates than subjects in treatment, whereas substance abuse treatment is associated with dramatically lower HIV seroconversion rates [17]. Finally, current evidence shows that the risk of developing antiretroviral resistance does not differ significantly between IDUs and non-IDUs [18]. These examples highlight the importance of comprehensive treatment programmes that address substance use and HIV concurrently to improve adherence and outcomes. Importantly, the concerns regarding the emergence of drug-resistant HIV strains have not materialised, even in jurisdictions that favour aggressive HAART treatment of substance users [19]. Furthermore, comprehensive HAART programmes targeting substance users are associated with substantial decreases in new HIV infections [20]. Accordingly, a new set of recommendations, put forth at a recent International AIDS Conference [21], emphasises the urgency of seeking (proactively identifying substance users), testing (annually, per US Centers for Disease Control and Prevention [CDC] recommendations), treating (using HAART, per current guidelines), and retaining (through optimal treatment of the substance use disorder) HIV-infected substance users.

The gap in the treatment of substance use disorders also cripples our efforts to combat the HIV epidemic by shrinking the reservoirs that persist among high-risk populations. An example is our failure to fully harness the unique therapeutic opportunities that exist among incarcerated individuals, more than half of whom have a history of substance use [22]. Indeed, of the over 200,000 individuals with heroin addiction who pass through American correctional facilities annually, only a fraction is offered opiate replacement therapy (ORT) during and after incarceration, and those facilities that do provide ORT limit it mostly to pregnant women [23]. As for HIV, currently there are no standardised practices regarding HIV testing and care across US prison and jail systems. This is a huge lost opportunity, for treatment of HIV-infected substance users in the criminal justice system could incorporate discharge planning and medication carryover to ensure continuity of care on reentry into the community; such structural changes could transform the face of the HIV epidemic.

The global gap is similarly disconcerting: in the five low-income and middle-income countries that contain nearly half of all IDUs infected with HIV, IDU access to ORT ranges from 0% (Russia) to 4% (China) [24]. This lack or inadequate implementation of ORT that flies in the face of its social, medical, and economic benefits [22] reflects both ideological opposition to ORT and also the difficulties of its implementation. For example, ORT in most countries requires daily oversight, which imposes an extra burden on an individual who may be required to spend several hours to access treatment while also trying to keep a job. Thus, alternative medication strategies that overcome these practical barriers would be valuable. For example, while current treatments for opiate addiction have focussed on agonist-mediated maintenance-detoxification (e.g. methadone or buprenorphine), there is also evidence that antagonists (i.e. naltrexone) can help achieve remission in heroin abusers [25]. Until recently the value of naltrexone had been limited by the very poor compliance with this medication. However, the development of an extended-release, injectable formulation of naltrexone that was recently approved by the US Food and Drug Administration (FDA) for the treatment of opioid addiction might help overcome the poor adherence observed with opioid antagonists. Although more research is needed, the data collected so far with this medication, which is administered only once a month, appear to support its efficacy for treating opioid addiction [26]. Another research goal in this context is the development of immunotherapies for the treatment of opiate addiction. The rationale behind such a vaccine is that stimulation of antibodies against heroin (and its active metabolites) could interfere with the drug’s entry into the brain, providing long-lasting protection against relapse in heroin addiction. Promising results from a preclinical report established proof of principle by showing that the generation of a robust and specific polyclonal antibody response in vaccinated rats was accompanied by significant reductions in both the antinociceptive effects of heroin and in the acquisition of heroin self-administration [27].

Substance abusers, and IDUs in particular, are presently much less likely to receive HAART, despite their higher rates of HIV infection [21]. This gap, if unaddressed, is shaping up as one of the most deleterious roadblocks to progress in the intersecting fields of substance use disorders and HIV. It is worth mentioning in this context that the fear that drug users might further increase their high risk behaviours while on HAART has not been substantiated; in fact, a number of studies have shown that providing HAART to IDUs was not associated with increased sexual or drug-related risk behaviours [28, 29]. Hence, one of the clearest messages to come out of the recent International AIDS Society Conference was that “treatment as prevention” is no longer a testable hypothesis but has now progressed to the realm of an urgent implementation priority [21].

In short, our efforts to improve public health outcomes *vis á vis* the HIV epidemic should reflect the solid new understanding that a) injecting and non-injecting drug abusers can successfully undergo HIV treatment; b) many substance abusers adhere to antiretroviral therapy as well as people who do not inject or take drugs; and c) injecting drug users who undergo substance abuse treatment are more likely to obtain and stay in treatment for their HIV infection. The combined evidence makes a compelling case for integrating substance abuse treatment with HIV treatment programmes and for providing substance abusers with universal access to HIV treatment. At the moment, the most effective strategy for advancing this agenda appears to be a focussed “seek, test, treat, and retain” effort to aggressively seek out high-risk, hard-to-reach substance abusers, offer them HIV testing and access to HIV treatment when medically appropriate, and provide the necessary support to help them stay in treatment – both for HIV as well as substance abuse [30]. Achieving these goals, however, will require structural changes in the health care system aimed at overcoming lingering obstacles that have inhibited the merging of substance abuse treatment with HIV programs for far too long. 

## ABBREVIATIONS

AIDS: = Acquired Immune Deficiency Syndrome
CDC: = (United States) Centers for Disease Control and Prevention
DA: = Dopamine
FDA: = United States Food and Drug Administration
HAART: = Highly Active Antiretroviral Therapy
HIV: = Human Immunodeficiency Virus
IDU: = Injection Drug Users
MA: = Methamphetamine
NAc: = Nucleus Accumbens
ORT: = Opiate Replacement Therapy
US(A): = United States (of America)
